# Discovery of YopE Inhibitors by Pharmacophore-Based Virtual Screening and Docking

**DOI:** 10.1155/2013/640518

**Published:** 2013-10-21

**Authors:** Gizem Ozbuyukkaya, Elif Ozkirimli Olmez, Kutlu O. Ulgen

**Affiliations:** Department of Chemical Engineering, Boğaziçi University, 34342 Istanbul, Turkey

## Abstract

Gram-negative bacteria *Yersinia* secrete virulence factors that invade eukaryotic cells via type III secretion system. One particular virulence member, *Yersinia* outer protein E (YopE), targets Rho family of small GTPases by mimicking regulator GAP protein activity, and its secretion mainly induces cytoskeletal disruption and depolymerization of actin stress fibers within the host cell. In this work, potent drug-like inhibitors of YopE are investigated with virtual screening approaches. More than 500,000 unique small molecules from ZINC database were screened with a five-point pharmacophore, comprising three hydrogen acceptors, one hydrogen donor, and one ring, and derived from different salicylidene acylhydrazides. Binding modes and features of these molecules were investigated with a multistep molecular docking approach using Glide software. Virtual screening hits were further analyzed based on their docking score, chemical similarity, pharmacokinetic properties, and the key Arg144 interaction along with other active site residue interactions with the receptor. As a final outcome, a diverse set of ligands with inhibitory potential were proposed.

## 1. Introduction

The Rho family of small (20–40 kDa) GTPases, which are monomeric G-proteins, belong to the Ras superfamily of GTPases containing more than 150 proteins [1]. The Ras superfamily has been classified into 5 subfamilies based on their sequence similarity which are Ras, Rho, Rab, Ran, and Arf [1, 2]. The Rho family of GTPases are cell membrane-associated GTP-binding proteins that actively participate in cell signaling networks, which regulate actin organization, cell cycle progression and gene expression [3, 4]. Up to date, 20 members of Rho GTPases have been found in four main subclasses, namely, Rho, Rnd, Rac, and Cdc42 [2, 5]. Rho GTPases are the most fundamental regulators of the actin cytoskeleton, along with other crucial properties in the cell, such as cell adhesion, gene transcription and cell proliferation, cell motility, vesicular trafficking, phagocytosis, and cytokinesis [2, 4–9].

Similar to other G-proteins and GTPases, Rho family proteins can serve as molecular switches, by binding to either GDP or GTP. GTPases are active and are capable of transmitting cell signals to downstream proteins when they are bound to GTP and inactive when they are bound to GDP [10, 11]. Since nucleotide association and dissociation are normally slow, some regulators within the cell catalyze the process of cycling between GDP- and GTP-bound states of Rho GTPases [12, 13]. These regulators are guanine nucleotide exchange factors (GEFs), GTPase-activating proteins (GAPs), and guanine nucleotide dissociation inhibitors (GDIs) [14]. GEFs stimulate the substitution of GDP for GTP to activate Rho GTPases, whereas GAPs inactivate Rho GTPases by stimulating the substitution of GTP for GDP. GDIs avoid the dissociation of GDP from GTPases and retain them in their nonsignaling state [15, 16]. Nucleotide exchange occurs due to the conformational changes in Switch I and Switch II regions of GTPases, upon their contact with GEFs/GAPs [5, 11]. 

Owing to the important role of Rho GTPases in cell signaling events and several cellular functions, they are favored by many bacterial pathogens as targets to deliver cytotoxins [18]. Bacterial effector proteins invade host cells via a specialized secretion system and regulate Rho GTPases by mimicking either GEF or GAP activity [10, 19]. Examples of such bacteria are *Burkholderia*, *Chlamydia*, *Salmonella*, *Shigella,* and *Yersinia* [18]. These bacteria use type III secretion system to inject their effector proteins directly into the host cell via a needle complex extending from the bacterial membrane and cytosol [20]. Bacterial proteins disorganize the actin cytoskeleton by depolymerization of actin stress fibers of the host cell. By rearranging actin dynamics, they disrupt cell shape and motility, phagocytosis, and cell division. Additionally, bacterial effector proteins can manipulate GTPase signaling mechanism and can transmit signals to downstream effector proteins [21, 22]. As a result, bacterial effector proteins can lead to many diseases, including infection and cancer [23]. YopE also has been reported to weaken the immune system of the host cell by affecting cytokine production and cause bubonic plague [24]. 

**Figure 1:**
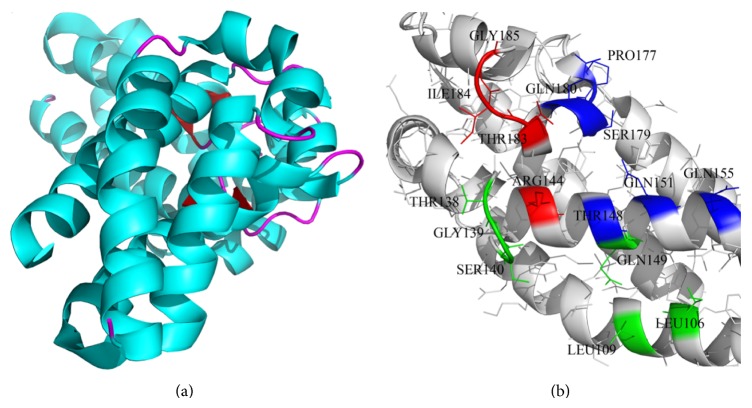
(a) Cartoon representation of YopE generated by Pymol [17]. (b) YopE residues are shown in stick, and the residues involved in G-protein interactions are labeled and colored. Switch I and Switch II interacting residues are indicated with green and blue, respectively, whereas red residues interact with both regions.

In this work, the bacterial protein toxin *Yersinia* outer protein E (YopE), which has been found to exert GAP activity towards RhoA, Rac1, and Cdc42 of GTPases in vitro, is investigated. [25–27]. YopE has been reported to disrupt actin cytoskeleton, prevent phagocytosis, and weaken host cell's immune system by affecting cytokine production [28–31]. YopE, shown in Figure 1, is a monomeric protein of 219 amino acids, with a four antiparallel *α*-helix bundle, four small helices, and two *β*-strands [24]. C-terminal domain of YopE between residues 90–219 is essential for its virulence since it comprises the bacterial GTPase activating protein (GAP) domain. Similar to other GAP proteins, YopE has an arginine residue (Arg144) that is reported to be essential for GAP activity [1]. In addition to Arg144, residues 182–184 are reported to be conserved among other bacterial GAP proteins, which are ExoS of *Pseudomonas aeruginosa* and SptP of *Salmonella *spp. (22% sequence identity with ExoS_GAP_ and 29% sequence identity with SptP_GAP_) [24]. YopE interactions with G-proteins are investigated and important YopE residues that govern its activity are reported by several studies [1, 3, 32]. Residues Ile106, Leu109, Thr138, Gly139, Ser140, and Gln149 are observed to interact with Switch II region of the GTPases. The key residue Arg144 along with Thr183, Ile184, and Gly185 is reported to contact the nucleotide and both of the switch regions. Residues Thr148, Gln151, Gln155, Pro177, Ser179, and Gln180 are found to interact with nucleotide and Switch I region of Rho GTPases [24].

Up to date, no cocrystallized or experimentally discovered ligands of YopE have been reported. However, inhibition of type III secretion system has been investigated by several studies [33–37], and 23 compounds that belong to a class of acylated hydrazones of different salicylaldehydes that prevent YopE secretion in vitro have been identified [38]. Salicylidene acylhydrazides, which have been denoted as a class of antivirulence compounds, were also reported to obstruct type III secretion of other Gram-negative bacteria other than *Yersinia* [39]. Although there were a number of mechanisms postulated, the inhibition mechanism and the target proteins of these salicylidene acylhydrazides were the focus of several studies [38, 40, 41]. Recently, *Yersinia pseudotuberculosis* proteins Tpx and WrbA, which take role in YopE secretion,were recognized as targets of these compounds, [39]. Nevertheless, other potential targets of salicylidene acylhydrazides remain unknown, whose identification is essential for the investigation and design of new therapeutic molecules against bacterial secretion mechanism.

Here, we represent a hybrid virtual screening approach to identify molecules with inhibition potential against YopE, using computational drug discovery tools. Pharmacophore building was carried out via Phase, based on the three-dimensional structures of 23 salicylidene acylhydrazides [38]. Small molecules selected from ZINC database were screened and filtered with the pharmacophore model. Multistep docking of filtered molecules was carried out with Glide, taking ligand flexibility into account. Virtual screening hits were clustered and further evaluated for their interactions with the target and pharmacokinetic properties. A set of commercially available molecules with possible inhibitory activity toward YopE was reported.

## 2. Methods

All virtual screening applications were performed using Schrödinger Suite 2011 (LLC, New York, NY, USA) on Linux platform using HP xw6600 Workstation. The following Schrödinger modules were used: Protein Preparation Wizard [42–44], LigPrep [45], ConfGen [46], Phase [47], SiteMap [48], QikProp [49], and Glide [50–52]. All modules were accessed via Maestro graphical interface [53]. 

### 2.1. Database Generation

The 3D structures of the small molecules from the big vendors of ZINC database (see Table S1 in Supplementary Material available online at http://dx.doi.org/10.1155/2013/640518) were prepared via LigPrep and ConfGen scripts and merged using Phase Database Generation module. The parameters used in the preparation step are the following: (a) possible ionization and tautomer states were generated at pH 7.0 ± 2.0; (b) chiralities were obtained from the 3D geometry of the structures; (c) for each compound, maximum 4 low energy isomers, 1 ring conformation, and 10 conformations per rotatable bond were generated; (d) 50 steps of energy minimization were carried out for the conformers with the truncated Newton (TCNG) method using the OPLS_2005 force field and a distance dependent dielectric constant of 4; (e) conformers with high-energy ionization/tautomer states were automatically removed and the number of conformers was limited to 100 per compound, which is the default value for conformer generation in Phase Database Generation module; (f) a built-in QikProp script predicted the ADME and druglikeness properties of the molecules; (g) molecules that violated Lipinski's rule (molecular weight <500, hydrogen bond donor <5, hydrogen bond acceptor <10, and partition coefficient <5) or had reactive functional groups were removed. Database preparation yielded a total of 25.8 million conformations from the initial 500000 molecules.

### 2.2. Receptor Protein Preparation

Docking studies were conducted on the target protein YopE. Coordinates of YopE (PDB id: 1hy5, [24]) were obtained from RCSB Protein Data Bank [54]. The biological assembly is known to be a monomer, and therefore, one YopE chain from the crystal dimer structure was prepared and refined using the Protein Preparation Wizard. Charges and bond orders were assigned, hydrogens were added to the heavy atoms, selenomethionines were converted to methionines, and all waters were deleted. Reorientation of certain hydroxyl and thiol groups, amide groups of asparagines, glutamines and imidazole ring of histidines, protonation states of histidines, aspartic acids, and glutamic acids was optimized at neutral pH. Using force field OPLS_2005, minimization was carried out setting maximum heavy atom RMSD to 0.30 Å.

### 2.3. Receptor Grid Generation

After preparation, receptor grids were generated with Glide by specifying the binding site with a 3D cubic box. SiteMap was used to estimate the location of the active site by searching regions near the protein surface, generating hydrophobic and hydrophilic contour maps of the protein, and calculating energy potentials. Enclosing box of 14 Å side lengths was placed by centering the critical Arg144 residue, depending on the SiteMap prediction (Figure S1). Based upon the fact that the binding site is not shallow, the nonpolar atoms were slightly scaled back, by choosing the van der Waals radius scaling factor of 0.75 for nonpolar parts, so that nonnative ligands would dock to the receptor better. Rotation of all receptor hydroxyl and thiol groups within the grid was allowed.

### 2.4. Ligand-Based Pharmacophore Generation

Pharmacophore model was developed using the 23 experimentally determined inhibitors of YopE secretion (Table 1) via Phase module. The small molecules from the literature were drawn in Maestro workspace, and these 2D structures were converted to all-atom 3D structures using embedded LigPrep script. Up to 32 stereoisomers were generated per ligand by determining chiralities from 3D structures, and all possible ionization states of ligands were generated at target pH of 7. After converting to 3D, conformation search was carried out to generate conformers and search for low energy structures using OPLS_2005 force field and other default parameters with ConfGen script. Maximum number of conformers per rotatable bond and number of conformers per structure were kept at 100 and 1000, respectively. Each compound, along with its different states and conformers, was represented by a set of points in space. When these set of points were aligned, some of them were found to coincide with each other, which indicates a structural feature or pharmacophore site. Six built-in sets of pharmacophore features were searched: hydrogen bond acceptor (A), hydrogen bond donor (D), hydrophobic group (H), negatively charged group (N), positively charged group (P), and aromatic ring (R). A common set of pharmacophore features that are observed consistently in inhibitors with similar spatial arrangements were identified and grouped. Then, all groups were individually investigated and tree-based partitioning technique was applied. If the grouped pharmacophore points do not coincide with at least one arbitrary pharmacophore site of each compound, they were eliminated. The remaining pharmacophore groups, which then are called pharmacophore hypotheses, were scored according to their alignment to the input molecules. Overall quality of each hypothesis was measured from its survival score, and AAADR.21 was selected. The database was filtered based on its 3D similarity to the selected pharmacophore hypothesis, such that the minimum fitness value was 1.4.

### 2.5. Glide Docking

Molecules obtained by filtering were used for multistep receptor docking workflow. Glide standard precision (SP) docking was performed with these molecules, and hits above 4 kcal/mol based on docking score were redocked to YopE in XP mode, keeping all docking parameters as default. No bonding constraints were given during docking calculations. Using Monte Carlo random search algorithm, ligand poses were generated for each input molecule, and binding affinity of these molecules to YopE was predicted in terms of Glide docking score. Potential energies of the docked molecules were also predicted with empirical E-model scoring function. Postdocking minimization was performed with OPLS_2005 force field, and one pose per ligand was saved. Strain energies of bound and free forms of ligands were calculated, and hits with more than 4 kcal/mol energy difference between the two forms received a penalty equal to the quarter of their strain energy difference, which is added to the docking score.

## 3. Results and Discussion

Investigation of novel inhibitors targeting the bacterial YopE was performed with the help of computational drug design tools. Database generation, pharmacophore modeling and screening, and molecular docking and scoring were carried out to propose a set of biochemically active molecules with inhibition potential against YopE.

### 3.1. Pharmacophore Development

23 chemically synthesized compounds belonging to a class of acylated hydrazones of salicylidene acylhydrazides that inhibit YopE secretion in vitro were utilized (Table S2) for pharmacophore development. The ability of these salicylidene acylhydrazides to inhibit YopE and to make important interactions upon direct contact, similar to Tpx and WrbA, was sought. For this purpose, prior to pharmacophore development, extra precision Glide docking was carried out with these 23 compounds, where docking scores were observed to be between −2.0 and −5.9 kcal/mol (data not shown). The binding orientations of these compounds were found to be in close proximity to the critical arginine residue, occupying the cleft between Arg144 and a bulge formed by residues Thr183-Gly185. The most common interactions between YopE and 23 inhibitors were observed in residues Arg144, Gln151, and Thr183. The compounds also interacted with other important residues reported upon GTPase contact, some of which are Thr138, Gly139, Thr148, Ser179, Gln180, Gly182, Ile184, and Gly185. The binding modes and numerous interactions between YopE and the salicylidene acylhydrazides supported the choice of using these compounds for pharmacophore development. Six out of 23 compounds that have the critical Arg144 interaction and docking scores above −4 kcal/mol were selected (Table 1). It was visually observed that, below this score, the likelihood of encountering favorable interactions diminishes. Interaction with the Arg144 of YopE was regarded as necessary since this residue was shown to interact with both of the switch regions of G-proteins [24], and its importance in catalytic activity was reported by mutation analyses [27, 55]. It was observed that Arg144 made hydrogen bonds either with the formic hydrazide group (NNC=O) or the hydroxybenzene groups of the compounds.

The pharmacophore model was developed based on the structures of the six selected compounds using Phase. After hypotheses were constructed, they were scored and ranked according to their coordination to the ligands, which can be seen in Table 2. A total of 20 variant hypotheses were generated upon pharmacophore development process.

**Figure 2:**
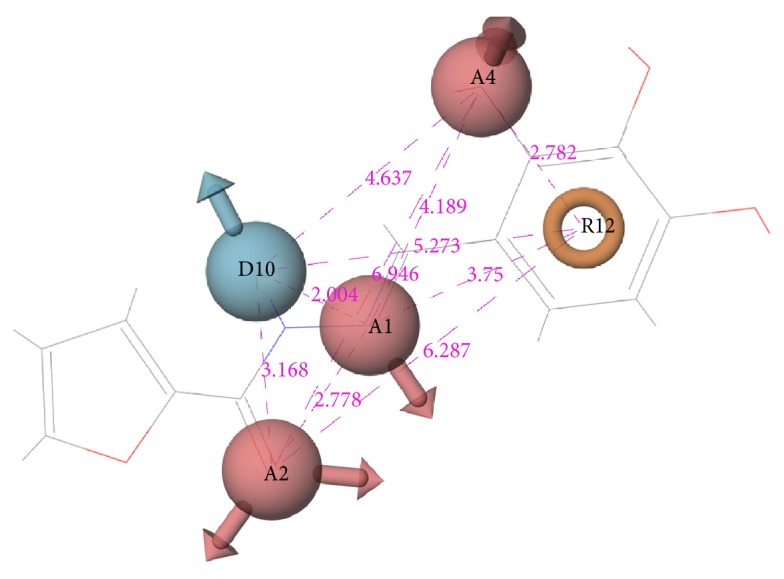
AAADR.21 site points and site-site distances on compound 15.

The quality of each hypothesis was measured by its survival score, which is a weighted combination of site, vector, volume, and selectivity scores. Site score indicates root mean squared deviation of compounds from the hypothesis site positions, whereas vector score is the average cosine of the angles formed by corresponding pairs of site features in the aligned structures and volume score measures how a compound overlays with pharmacophore sites based on van der Waals spheres. Site, volume, and vector scores range between 0 and 1, and high values indicate better alignment of ligands on hypothesis. Selectivity is an empirical prediction defined on log scale, which calculates the fraction of molecules that could match the hypothesis, whether it is an active or inactive ligand. For example, a selectivity value of 2 means that 1 molecule out of 100 (log 10^2^) arbitrary ligand molecules would match the hypothesis, regardless of their activity value. Therefore, higher selectivity is favored, since it implies uniqueness to the ligand set. However, site, vector and, volume scores are underlined in the literature due to their geometric significance [56]. Overall, five-point hypothesis AAADR.21, with three hydrogen acceptors, one hydrogen donor, and one ring group, gave the best three-dimensional alignment to the selected six compounds in terms of site, volume, selectivity, and total survival scores. AAADR.21 site points matched with compound 15 are represented in Figure 2 along with pharmacophore site-site distances. Two hydrogen acceptors and one hydrogen donor were observed to coincide with the atoms of Arg144 interacting formic hydrazide group, whereas the other acceptor was aligned with the hydroxyl group of the benzene ring. Site distances were also found to be reasonable. Site angle measurements are given in Table S3. Pharmacophore model was used to search 3D database (small molecule database generated from ZINC) to identify the molecules that satisfy the hypothesis. Pharmacophore prefiltering with AAADR hypothesis reduced initial 2.5 million conformers to 18230 hits.

### 3.2. Molecular Docking and Hit Selection

The molecules obtained by filtering with hypothesis AAADR.21 were docked to the receptor YopE with Glide software to predict binding affinity of molecules to the target and to investigate their ligand-protein interactions. The binding orientation and features of each database molecule relative to the receptor protein were determined and scored with internal scoring function GlideScore. Glide standard precision (SP) docking was first conducted with 18230 hits using the 3D structure of YopE prepared for docking calculations, as described in the receptor protein preparation and receptor grid generation sections. Top 30% hits of Glide SP in terms of docking score, which comprises 5604 molecules, were redocked to YopE in Glide extra precision (XP) mode with the identical internal docking parameters. Docking scores varied between −7.2 and −0.5 kcal/mol in Glide XP mode. A pose filter was carried out and virtual screening hits that interact with the critical Arg144 residue were determined. This filter yielded 2605 hits out of the initial 5604. The number of remaining hits was further reduced to 185 by filtering with a docking score threshold of −4 kcal/mol. Structures of the remaining molecules were analyzed and clustered by their 2D structural similarities via ChemMine Web Tool [57]. Hierarchy of clusters based on pairwise compound similarity was defined using the Tanimoto similarity coefficient and structural descriptors such as atom pairs. The hierarchical similarity tree output is provided in Figure S2. The hits were clustered using the Tanimoto coefficient threshold as 0.50. Based on this similarity criterion, the majority of the hits formed individual cluster or only small groups of two or three members. Only three more populated groups, with 8, 26, and 11 members, were formed according to the ChemMine similarity clustering. Database titles and docking scores of each member of these large clusters are tabulated in Table S4. Three top scoring members of aforementioned clusters were chosen for visual inspection and detailed analysis. The 2D structures and docking results of the hits are given in Tables 3 and 4. The docking scores of the selected hits were found to be higher than the initial six compounds that were used in pharmacophore development. Three molecules from each cluster were analyzed based on the docking score as well as additional criteria, such as absorption, distribution, metabolism and excretion (ADME) considerations, druglikeness of ligands, and strain energy differences of ligands.

Molecules were sorted according to their docking scores in Table 4. Detailed docking score components of each molecule are also listed in Table S5. Docking calculations were performed keeping the protein structure rigid and ligands flexible. Therefore, ligands were allowed to be strained during docking, in order to fit the ligands to the protein binding site. However, too much strain may indicate false positives, and therefore, the strain in the molecules were determined. To this end, energies of free and docked conformations of hits were calculated and strain penalties were determined as a postdocking analysis, as described in Glide docking section. Another scoring function represented in Table 4, namely, E-model, also includes penalty terms for internal strain energy of the generated poses. Only ZINC01703513, ZINC19800113, and ZINC05297691 received a small strain penalty, which was considered as insignificant.

Pharmacokinetic properties, druglikeness as well as other significant descriptors, such as molecular weight, H-bond donors, H-bond acceptors, solvent accessible surface area, log HERG (blockage of K+ channels), log S (aqueous solubility), log P (octanol/water), and human oral absorption, for the selected hits were determined by QikProp (Table 5). Druglikeness, as predicted by the Lipinski rule, was investigated along with the predicted ADME and molecular properties. According to this rule, in order for a compound to be drug-like and orally active, it should have a molecular weight less than 500 Da, hydrogen bond donor equal to or less than five, hydrogen bond acceptor equal to or less than 10, and partition coefficient (QP log Po/w) less than five. Molecular weight, donor and acceptor atom numbers of the selected molecules were within the allowed values. QP log Po/w (or Log P) gives an estimate of compound's lipophilicity. Up to a certain limit, compounds with higher lipophilicity have higher ability to permeate across biological membranes, which is necessary for a drug candidate. In this study, all nine hits had acceptable values for the analyzed properties and exhibited drug-like characteristics based on the rule of 5. The complete list of predicted physiochemical descriptors and ADME properties is given in Table S6.

### 3.3. Binding Mode Analysis and Visual Inspection of the Selected Hits

**Figure 3:**
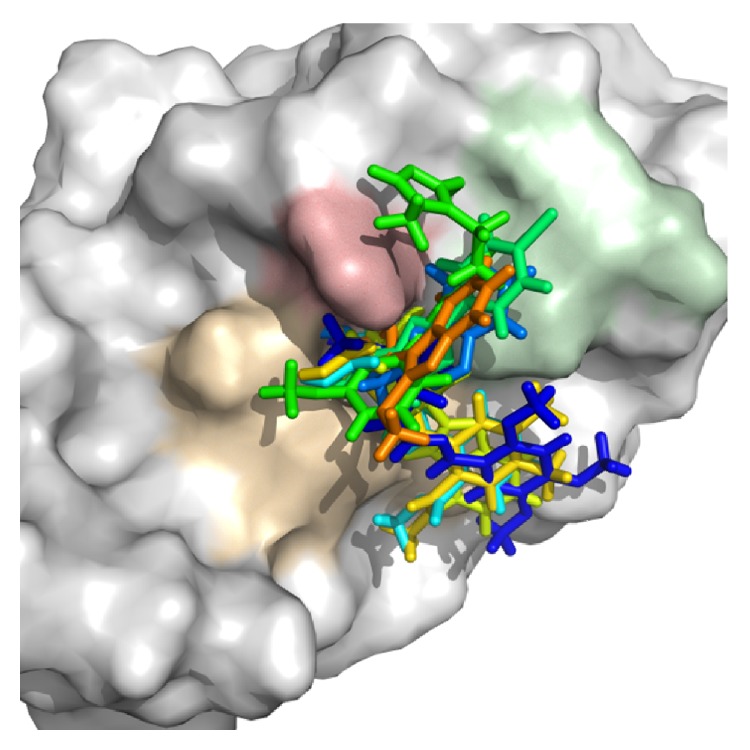
Superimposition of the selected hits on the YopE binding site. YopE is represented as surface, and ligands are shown as sticks. Arg144 residue is indicated with red, Gly182, Thr183, and Ile184 are in green, and Thr148 and Gln151 are in light brown.

**Figure 4:**
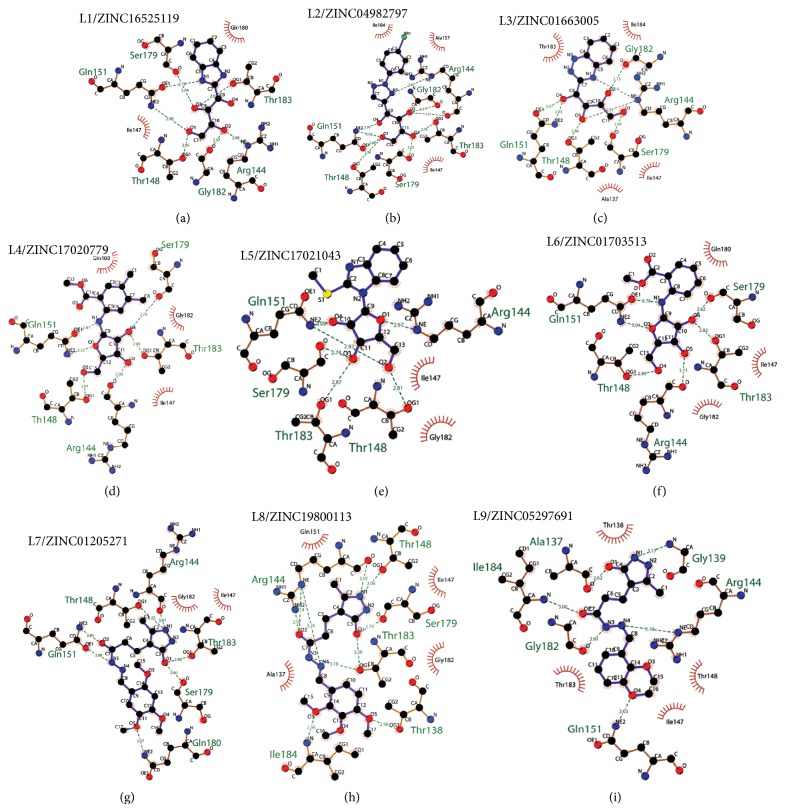
Ligand-protein interaction diagrams of the selected hits generated by LIGPLOT [58].

Superposition of the proposed molecules YopE showed that their binding orientations are similar (Figure 3). The interactions of the nine selected hits (L1–L9, Table 4) were analyzed to verify that the ligands made contacts with previously identified important residues of YopE. All of the selected hits were found to reside in the cavity surrounded by the critical Arg144 residue and other residues that are known to react with the switch regions of the G-proteins. The 2D representation of the selected hits and their receptor hydrogen bond and hydrophobic interactions is shown in Figure 4. The ligand-protein interaction diagrams were generated in LIGPLOT [58] by supplying the receptor-ligand complexes to PDBsum [59] in pdb format. Hydrogen bond interactions and their atomic distances (in Å) are shown in dashed lines, whereas hydrophobic contacts are shown in red crescents. All proposed molecules favoring the hydrogen bond with critical Arg144 residue, which is known to be essential for GAP function of YopE. L1, which has the highest docking score, had multiple hydrogen bond interactions with YopE residues that interact with the switch regions in addition to Arg144. These residues are Thr148, Gln151, Ser179, Gly182 and Thr183. Gln151 and Ser179 are known to bond with both nucleotide and Switch I region of Rho GTPases, whereas Thr183 interacts with the two switch regions. Similar interactions were also observed in the majority of hits, which can be seen in Figure 4. Fewer interactions were made with Ala137, Gly139, Gln180, and Ile184 of YopE. Gly139 and Gln180 are also among the reported Switch I interacting residues, and Ile184, similar to Thr183, has the ability to make contacts with both of the switch regions. Multiple hydrophobic contacts were also observed in each ligand-protein complex, with Ile147 and Gly182 being the most significant. Overall, the numbers of bonded interactions and hydrophobic contacts were observed to be high, suggesting a strong binding between the proposed hits and the target protein. In addition, the binding orientation of the hits was observed to occlude the cleft comprising Arg144 and other switch-interacting residues, and hence, presence of these compounds could possibly block the interaction between YopE and GTPases.

The hits were visually inspected and their similarity to the salicylidene acylhydrazides used in pharmacophore development was determined. The nine selected hits satisfied all of the pharmacophore sites with a 2 Å tolerance, suggesting that the pharmacophore building and docking results converged. The superposition of the selected hits on the pharmacophore hypothesis AAADR.21 is given in Figure S3. The hits from the third cluster (Table 4) were found to overlay very well with the pharmacophore sites. These hits also showed close structural resemblance to each other as well as to the initial six salicylidene acylhydrazides. Members of the third cluster, L7, L8, and L9, include a 5-hydroxypyrazole ring in their structure. Similar to salicylidene acylhydrazides, they also have two ring conformations on the sides connected to a formic hydrazide group (NNC=O). The ligand interaction maps reveal that the formic hydrazide substructure has multiple contacts with the YopE binding site residues (Figures 4(g), 4(h), and 4(i)). The first two clusters, on the other hand, do not share a notable structural similarity with salicylidene acylhydrazides although they align with pharmacophore site points to a certain extent. The first cluster, which includes L1, L2, and L3, has a common erythritol (R-butane-1,2,3,4-tetraol) substructure (Figures 4(a), 4(b), and 4(c)), whereas hits from the second cluster, L4, L5, and L6, have tetrahydrofuran-2,3,4-triol ring in their structure (Figures 4(d), 4(e), and 4(f)). Both of these substructures also account for the vast majority of interactions between YopE and ligands. Hence, the scaffolds that have been observed within each cluster may lead to the indication of potent functional groups upon YopE binding.

### 3.4. Selectivity

The *P. aeruginosa* cytotoxins ExoS_GAP_ [13, 60] and *S. enterica *SptP_GAP _ [61] are the homolog GAP proteins of YopE with 22% and 29% amino acid sequence identity, respectively. Although their sequence identity is remarkably low, their structure alignment on the backbone shows considerable resemblance. After superposition based on the YopE C_*α*_ coordinates, the root mean square deviations between the C_*α*_ coordinates of YopE with ExoS_GAP_ and with SptP_GAP_ are 1.26 Å and 1.36 Å, respectively [24]. The key arginine finger of YopE (Arg144) was also conserved in these GAP proteins, as Arg146_ExoSGAP_ and Arg209_SptPGAP_. Binding selectivity of selected hits to ExoS_GAP_ and SptP_GAP_ was investigated. Their 3D structures were taken from the Protein Data Bank (Pdb ID: 1g4u for SptP_GAP_ and Pdb id: 1he1 for ExoS_GAP_) prepared in Maestro workspace, and their binding site grids were generated for all-atom docking calculations, as described in receptor protein preparation and receptor grid generation sections. By centering the key arginine residues and keeping the previous docking parameters identical, Glide XP docking was performed on ExoS_GAP_ and SptP_GAP_. The results reveal that the selected nine hits have higher average ligand strain and lower binding affinity toward ExoS_GAP_ and SptP_GAP_ in terms of docking scores (Table 6). Furthermore, only L2 and L7 interacted with Arg146 of ExoS_GAP_ and only L5 interacted with Arg209 of SptP_GAP_. The absence of the critical arginine interaction as well as the low scores suggests that the proposed molecules may not bind the homologous proteins and that they are selective toward YopE_GAP_.

## 4. Conclusions

In this work, the aim was to discover small molecules with inhibition potential against YopE, which is a bacterial cytotoxin that inhibits small Rho GTPases by mimicking their regulator proteins within the host cell. Proper functioning of GTPases is crucial for the regulation of signaling events within the cell, and therefore, drug design against GTPase inhibitors, such as YopE, is an important area of research. Here, we used virtual screening to investigate potent drug-like inhibitors of YopE. 23 small compounds, which were previously shown to inhibit the YopE secretion mechanism, were utilized to develop a pharmacophore hypothesis. 500,000 unique small drug-like molecules selected from the ZINC database were filtered based on 3D similarity to the hypothesis AAADR. Binding orientations and features of these molecules were investigated with multistep molecular docking approach using Glide software, allowing ligand flexibility. 

Virtual screening hits that exhibit a certain binding affinity to YopE in terms of docking score (−4 kcal/mol) were clustered based on their structural similarity using a Tanimoto coefficient threshold of 0.5. The three top scoring representative members from the most populated clusters were pooled and further analyzed. One cluster was found to be structurally similar to the salicylidene acylhydrazides, which can inhibit the bacterial activity of type III secretion systems. The cluster with erythritol substructure is analogous to THI (2-acetyl-4-tetrahydroxybutylimidazole). THI acts as an immunosuppressant inhibitor of Sphingosine-1-phosphate lyase (S1PL), whose reduced activity is targeted for autoimmune disorder treatment [62]. The other cluster, having tetrahydrofuran-2,3,4-triol ring as a common substructure, shares similarity to Nelarabine, a drug used in the treatment of T-cell lymphoblastic leukemia [63]. Druglikeness of the clusters was also predicted, and molecular descriptors and pharmacokinetic and ADME properties of the nine hits were found to be in accordance with known chemically and biologically active compounds. 

The aim of this virtual screening study was to find potent YopE inhibitors that would hinder the interaction between their GAP domain and GTPases. The binding site was selected such that the inhibitors would occlude the vicinity of the Arg144 residues that contact the switch regions of GTPases. Indeed, the molecules made contacts with the critical arginine finger as well as the other residues that were reported to interact with both Switch I and Switch II regions. The binding modes of the hits showed that the molecules occupied the cleft formed in the vicinity of Arg144 of YopE. Selectivity against YopE was verified by docking the nine hits to the known YopE homologs, namely, ExoS_GAP_ and SptP_GAP_. Docking results showed that these hits have lower binding affinity toward the homologous proteins, and the critical arginine bonding was not observed in the majority of hits. The proposed set of ligands has shown a promising inhibitory potential toward YopE *in silico* and hence, can be used for further scientific studies, and the results can be extended to experimental validation.

## Supplementary Material

The list of vendors used in small molecule database (Table S1), 2D structures of small compounds found in literature (Table S2), pharmacophore site measurements (Table S3), detailed docking results (Table S4 and Table S5), detailed ADME and pharmacokinetic property estimates (Table S6), SiteMap and ChemMine outputs (Figure S1 and Figure S2) and pharmacophore-hit superimpositions (Figure S3) are provided.

## Figures and Tables

**Table 1:** 2D structures of experimentally determined inhibitors utilized for pharmacophore development [38].

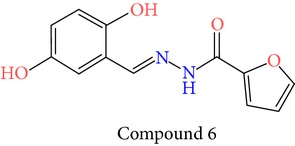	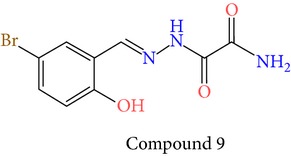	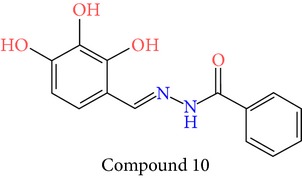
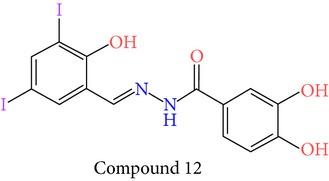	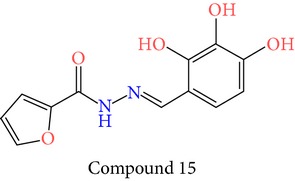	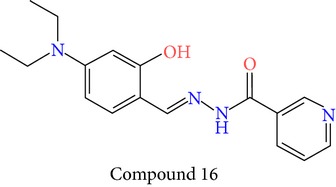

**Table 2:** Scores of pharmacophore hypotheses generated in Phase.

ID	Survival	Site	Vector	Volume	Selectivity	Number of Matches
AAADR.21	3.858	0.99	1.000	0.865	1.147	6
AAARR.87	3.759	0.95	0.953	0.859	1.467	5
AADRR.98	3.749	0.94	0.955	0.855	1.551	5
AAARR.81	3.738	0.92	0.949	0.864	1.456	5
ADDRR.61	3.726	0.91	0.950	0.865	1.643	5
AADDR.43	3.723	0.90	0.974	0.846	1.200	5
AADRR.23	3.719	0.91	0.948	0.858	1.457	5
AAARR.4	3.708	0.91	0.946	0.856	1.422	5
AADRR.46	3.707	0.94	0.926	0.838	1.488	5
AADDR.10	3.704	0.94	0.926	0.835	1.231	5
AADRR.127	3.437	0.72	0.892	0.821	1.591	5
ADDRR.67	3.416	0.74	0.888	0.791	1.624	5
AADDR.56	3.405	0.72	0.891	0.792	1.304	6
ADDRR.72	3.398	0.72	0.867	0.812	1.604	5
AADDR.58	3.396	0.73	0.865	0.803	1.329	5
AAAAR.5	3.072	0.50	0.858	0.709	1.320	5
ADDRR.9	2.997	0.56	0.791	0.645	1.601	5
AADRR.19	2.904	0.58	0.680	0.643	1.401	5
AADDR.11	2.593	0.35	0.705	0.537	1.282	5
AAADR.2	2.155	0.14	0.617	0.398	1.138	6

**Table 3:** 2D structures of selected hits with docking scores in kcal/mol.

Cluster 1	Cluster 2	Cluster 3
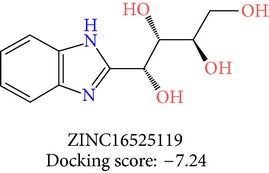	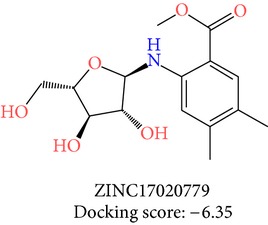	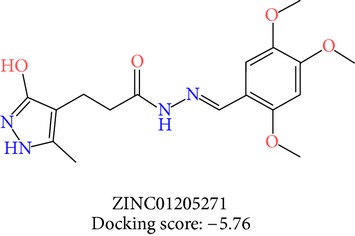
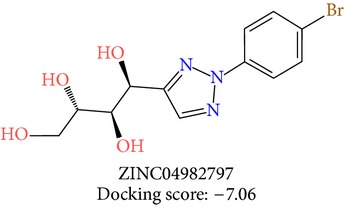	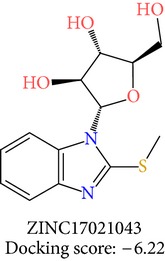	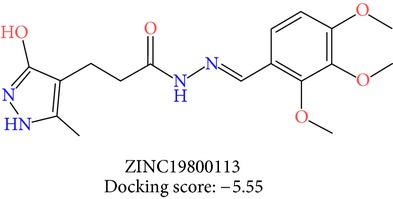
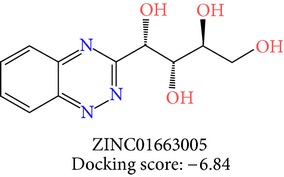	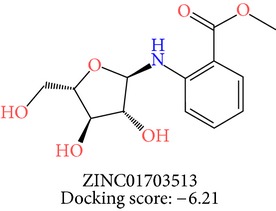	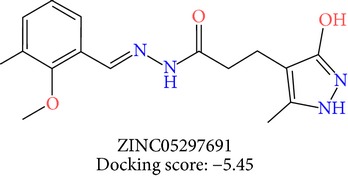

**Table 4:** Docking results of selected hits in kcal/mol.

Cluster	Name	Title	Initial Ranking	Docking score	Bound energy	Free energy	Strain energy	Strain penalty	Glide Emodel
1	L1	ZINC16525119	1	−7.24	23.77	22.08	1.68	0.00	−30.99
	L2	ZINC04982797	2	−7.06	22.14	19.71	2.43	0.00	−39.41
	L3	ZINC01663005	3	−6.84	34.19	30.26	3.94	0.00	−23.80

2	L4	ZINC17020779	6	−6.35	36.65	32.87	3.78	0.00	−32.71
	L5	ZINC17021043	7	−6.22	42.00	39.56	2.44	0.00	−27.33
	L6	ZINC01703513	8	−6.21	36.70	31.25	5.45	0.36	−32.82

3	L7	ZINC01205271	18	−5.76	22.82	21.50	1.32	0.00	−38.36
	L8	ZINC19800113	24	−5.55	35.08	30.08	5.01	0.25	−40.66
	L9	ZINC05297691	26	−5.45	22.84	17.43	5.41	0.35	−32.16

**Table 5:** ADME and druglikeness properties of the selected ligands by QikProp.

Name	Title^a^	MW^b^	HB donor^c^	HB acceptor^d^	SASA^e^	QP log HERG^f^	QP log S^g^	QP log Po/w^h^	% Human Oral Absorption^i^
L1	ZINC16525119	238.2	5	8.3	470.8	−4.616	−1.49	−0.866	60.0
L2	ZINC04982797	344.2	4	8.8	546.7	−5.136	−2.62	0.414	68.4
L3	ZINC01663005	251.2	4	9.8	481.9	−4.626	−1.40	−1.023	56.1
L4	ZINC17020779	311.3	3	8.8	573.6	−4.383	−2.79	0.626	72.0
L5	ZINC17021043	296.3	3	8.3	499.1	−4.110	−2.30	0.641	78.5
L6	ZINC01703513	283.3	3	8.8	502.5	−4.257	−1.82	0.055	67.8
L7	ZINC01205271	362.4	3	5.8	704.1	−5.648	−5.13	2.892	84.9
L8	ZINC19800113	362.4	3	5.8	680.7	−5.401	−4.74	2.797	84.2
L9	ZINC05297691	332.4	3	5.0	655.8	−5.619	−4.68	2.725	83.9

^a^ZINC IDs.

^b^Molecular weight (acceptable range: <500).

^c^Hydrogen bond donor (acceptable range: ≤5).

^d^Hydrogen bond acceptor (acceptable range: ≤10).

^e^Total Solvent Accessible Surface Area in Å^2^ using a probe with a 1.4 Å radius (acceptable range: 300–1000).

^f^Predicted IC50 value for blockage of HERG K+ channels (concern: below −5).

^g^Predicted aqueous solubility, S in mol/dm^−3^ (acceptable range: −6.5–0.5).

^h^Predicted octanol/water partition coefficient (acceptable range: −2–6.5).

^i^Predicted human oral absorption on 0 to 100% scale (<25% is poor and >80% is high).

**Table 6:** Glide XP results of selected hits docked to ExoS_GAP_ and SptP_GAP_. All values are shown in kcal/mol.

Name	Title	ExoS_GAP_			SptP_GAP_		
		Docking score	Strain penalty	Arg146 bonding	Docking score	Strain penalty	Arg209 bonding
L1	ZINC16525119	−5.09	0.00	−	−4.25	0.00	−
L2	ZINC04982797	−6.11	0.32	+	−5.30	0.00	−
L3	ZINC01663005	−6.23	0.00	−	−4.95	0.00	−
L4	ZINC17020779	−3.28	0.00	−	−4.59	0.00	−
L5	ZINC17021043	−2.87	3.00	−	−4.41	0.00	+
L6	ZINC01703513	−3.30	0.00	−	−5.63	0.00	−
L7	ZINC01205271	−3.11	0.00	+	−3.27	0.00	−
L8	ZINC19800113	−4.33	2.48	−	−4.66	1.86	−
L9	ZINC05297691	−4.48	2.85	−	−4.21	0.38	−

## Abbreviations

ADME: Absorption, distribution, metabolism, and excretion
YopE: *Yersinia* outer protein E
SP: Standard precision
XP: Extra precision.

## References

[1] Wennerberg K., Rossman K. L., Der C. J. (2005). The Ras superfamily at a glance. *Journal of Cell Science*.

[2] van Aelst L., D'Souza-Schorey C. (1997). Rho GTPases and signaling networks. *Genes and Development*.

[3] Brändén C. I., Tooze J. (1999). *Introduction to Protein Structure*.

[4] Etienne-Manneville S., Hall A. (2002). Rho GTPases in cell biology. *Nature*.

[5] Rossman K. L., Der C. J., Sondek J. (2005). GEF means go: turning on Rho GTPases with guanine nucleotide-exchange factors. *Nature Reviews Molecular Cell Biology*.

[6] Raftopoulou M., Hall A. (2004). Cell migration: Rho GTPases lead the way. *Developmental Biology*.

[7] Chimini G., Chavrier P. (2000). Function of Rho family proteins in actin dynamics during phagocytosis and engulfment. *Nature Cell Biology*.

[8] Kjøller L., Hall A. (1999). Signaling to Rho GTPases. *Experimental Cell Research*.

[9] Sah V. P., Seasholtz T. M., Sagi S. A., Brown J. H. (2000). The role of Rho in G protein-coupled receptor signal transduction. *Annual Review of Pharmacology and Toxicology*.

[10] Ellenbroek S. I. J., Collard J. G. (2007). Rho GTPases: functions and association with cancer. *Clinical and Experimental Metastasis*.

[11] Schmidt A., Hall A. (2002). Guanine nucleotide exchange factors for Rho GTPases: turning on the switch. *Genes and Development*.

[12] Scheffzek K., Ahmadian M. R., Wittinghofer A. (1998). GTPase-activating proteins: helping hands to complement an active site. *Trends in Biochemical Sciences*.

[13] Würtele M., Wolf E., Pederson K. J. (2001). How the *Pseudomonas aeruginosa* ExoS toxin downregulates Rac. *Nature Structural Biology*.

[14] Bishop A. L., Hall A. (2000). Rho GTPases and their effector proteins. *Biochemical Journal*.

[15] Ridley A. (2013). GTPase switch: Ras then Rho and Rac. *Nature Cell Biology*.

[16] Cherfils J., Zeghouf M. (2011). Chronicles of the GTPase switch. *Nature Chemical Biology*.

[17] *The PyMOL Molecular Graphics System, Version 1. 5. 0. 4*.

[18] Finlay B. B. (2005). Bacterial virulence strategies that utilize Rho GTPases. *Current Topics in Microbiology and Immunology*.

[19] Boettner B., van Aelst L. (2002). The role of Rho GTPases in disease development. *Gene*.

[20] Galán J. E., Collmer A. (1999). Type III secretion machines: bacterial devices for protein delivery into host cells. *Science*.

[21] Wang Y., Zhang L., Picking W. L., Picking W. D., de Guzman R. N. (2008). Structural dissection of the extracellular moieties of the type III secretion apparatus. *Molecular BioSystems*.

[22] Toksoz D., Merdek K. D. (2002). The Rho small GTPase: functions in health and disease. *Histology and Histopathology*.

[23] Sahai E., Marshall C. J. (2002). RHO—GTPases and cancer. *Nature Reviews Cancer*.

[24] Evdokimov A. G., Tropea J. E., Routzahn K. M., Waugh D. S. (2002). Crystal structure of the *Yersinia pestis* GTpase activator YopE. *Protein Science*.

[25] Andor A., Trülzsch K., Essler M. (2001). YopE of *Yersinia*, a GAP for Rho GTPases, selectively modulates Rac-dependent actin structures in endothelial cells. *Cellular Microbiology*.

[26] Black D. S., Bliska J. B. (2000). The RhoGAP activity of the *Yersinia pseudotuberculosis* cytotoxin YopE is required for antiphagocytic function and virulence. *Molecular Microbiology*.

[27] von Pawel-Rammingen U., Telepnev M. V., Schmidt G., Aktories K., Wolf-Watz H., Rosqvist R. (2000). GAP activity of the *Yersinia* YopE cytotoxin specifically targets the Rho pathway: a mechanism for disruption of actin microfilament structure. *Molecular Microbiology*.

[28] Fallman M., Andersson K., Hakansson S., Magnusson K.-E., Stendahl O., Wolf-Watz H. (1995). *Yersinia pseudotuberculosis* inhibits Fc receptor-mediated phagocytosis in J774 cells. *Infection and Immunity*.

[29] Grosdent N., Maridonneau-Parini I., Sory M., Cornelis G. R. (2002). Role of Yops and adhesins in resistance of *Yersinia enterocolitica* to phagocytosis. *Infection and Immunity*.

[30] Ruckdeschel K., Roggenkamp A., Schubert S., Heesemann J. (1996). Differential contribution of *Yersinia enterocolitica* virulence factors to evasion of microbicidal action of neutrophils. *Infection and Immunity*.

[31] Schotte P., Denecker G., van Den Broeke A., Vandenabeele P., Cornelis G. R., Beyaert R. (2004). Targeting Rac1 by the *Yersinia* effector protein YopE inhibits caspase-1-mediated maturation and release of interleukin-1*β*. *The Journal of Biological Chemistry*.

[32] Alberts B. (2002). *Molecular Biology of the Cell*.

[33] Kauppi A. M., Nordfelth R., Hägglund U., Wolf-Watz H., Elofsson M. (2003). Salicylanilides are potent inhibitors of type III secretion in *Yersinia*. *Advances in Experimental Medicine and Biology*.

[34] Kauppi A. M., Nordfelth R., Uvell H., Wolf-Watz H., Elofsson M. (2003). Targeting bacterial virulence: inhibitors of type III secretion in *Yersinia*. *Chemistry and Biology*.

[35] Muschiol S., Normark S., Henriques-Normark B., Subtil A. (2009). Small molecule inhibitors of the *Yersinia* type III secretion system impair the development of Chlamydia after entry into host cells. *BMC Microbiology*.

[36] Garrity-Ryan L. K., Kim O. K., Balada-Llasat J. (2010). Small molecule inhibitors of LcrF, a *Yersinia pseudotuberculosis* transcription factor, attenuate virulence and limit infection in a murine pneumonia model. *Infection and Immunity*.

[37] Hudson D. L., Layton A. N., Field T. R. (2007). Inhibition of type III secretion in *Salmonella enterica* serovar typhimurium by small-molecule inhibitors. *Antimicrobial Agents and Chemotherapy*.

[38] Nordfelth R., Kauppi A. M., Norberg H. A., Wolf-Watz H., Elofsson M. (2005). Small-molecule inhibitors specifically targeting type III secretion. *Infection and Immunity*.

[39] Wang D., Zetterström C. E., Gabrielsen M. (2011). Identification of bacterial target proteins for the salicylidene acylhydrazide class of virulence-blocking compounds. *The Journal of Biological Chemistry*.

[40] Veenendaal A. K. J., Sundin C., Blocker A. J. (2009). Small-molecule type III secretion system inhibitors block assembly of the *Shigella* type III secreton. *Journal of Bacteriology*.

[41] Tree J. J., Wang D., McInally C. (2009). Characterization of the effects of salicylidene acylhydrazide compounds on type III secretion in *Escherichia coli* O157:H7. *Infection and Immunity*.

[42] (2011). *Epik Version 2. 2*.

[43] (2011). *Impact version 5. 7*.

[44] (2011). *Prime version 2. 3*.

[45] (2011). *LigPrep, Version 2. 5*.

[46] (2011). *ConfGen, Version 2. 3*.

[47] (2011). *Phase, Version 3. 3*.

[48] (2011). *SiteMap, Version 2. 5*.

[49] (2011). *QikProp, Version 3. 4*.

[50] Halgren T. A., Murphy R. B., Friesner R. A. (2004). Glide: a new approach for rapid, accurate docking and scoring. 2. Enrichment factors in database screening. *Journal of Medicinal Chemistry*.

[51] Friesner R. A., Banks J. L., Murphy R. B. (2004). Glide: a new approach for rapid, accurate docking and scoring. 1. Method and assessment of docking accuracy. *Journal of Medicinal Chemistry*.

[52] Friesner R. A., Murphy R. B., Repasky M. P. (2006). Extra precision glide: docking and scoring incorporating a model of hydrophobic enclosure for protein-ligand complexes. *Journal of Medicinal Chemistry*.

[53] (2011). *Maestro, Version 9. 2*.

[54] Bernstein F. C., Koetzle T. F., Williams G. J. B. (1977). The protein data bank. A computer based archival file for macromolecular structures. *European Journal of Biochemistry*.

[55] Ahmadian M. R., Stege P., Scheffzek K., Wittinghofer A. (1997). Confirmation of the arginine-finger hypothesis for the GAP-stimulated GTP-hydrolysis reaction of Ras. *Nature Structural Biology*.

[56] Dixon S. L., Smondyrev A. M., Knoll E. H., Rao S. N., Shaw D. E., Friesner R. A. (2006). PHASE: a new engine for pharmacophore perception, 3D QSAR model development, and 3D database screening: 1. Methodology and preliminary results. *Journal of Computer-Aided Molecular Design*.

[57] Backman T. W. H., Cao Y., Girke T. (2011). ChemMine tools: an online service for analyzing and clustering small molecules. *Nucleic Acids Research*.

[58] Wallace A. C., Laskowski R. A., Thornton J. M. (1995). LIGPLOT: a program to generate schematic diagrams of protein-ligand interactions. *Protein Engineering*.

[59] Laskowski R. A., Hutchinson E. G., Michie A. D., Wallace A. C., Jones M. L., Thornton J. M. (1997). PDBsum: a web-based database of summaries and analyses of all PDB structures. *Trends in Biochemical Sciences*.

[60] Würtele M., Renault L., Barbieri J. T., Wittinghofer A., Wolf E. (2001). Structure of the ExoS GTPase activating domain. *FEBS Letters*.

[61] Stebbins C. E., Galán J. E. (2000). Modulation of host signaling by a bacterial mimic: structure of the *Salmonella* effector SptP bound to Rac1. *Molecular Cell*.

[62] Yu X. Q., Kramer J., Moran L. (2010). Pharmacokinetic/pharmacodynamic modelling of 2-acetyl-4(5)-tetrahydroxybutyl imidazole-induced peripheral lymphocyte sequestration through increasing lymphoid sphingosine 1-phosphate. *Xenobiotica*.

[63] Cohen M. H., Johnson J. R., Justice R., Pazdur R. (2008). FDA drug approval summary: nelarabine (Arranon) for the treatment of T-cell lymphoblastic leukemia/lymphoma. *Oncologist*.

