# Supplementary material for: Discovery of YopE Inhibitors by Pharmacophore-Based Virtual Screening and Docking
Source: ISRN Bioinform. 2013 Oct 21;2013:640518. doi: 10.1155/2013/640518 (PMC4393062; doi:10.1155/2013/640518)
Supplement: Supplementary file 1 — The list of vendors used in small molecule database (Table S1), 2D structures of small compounds found in literature (Table S2), pharmacophore site measurements (Table S3), detailed docking results (Table S4 and Table S5), detailed ADME and pharmacokinetic property estimates (Table S6), SiteMap and ChemMine outputs (Figure S1 and Figure S2) and pharmacophore-hit superimpositions (Figure S3) are provided. [file 640518.f1.docx]

**Supplementary Material**

**Table S1:** Names of used vendors obtained from ZINC small molecule database (Access Date: 09/10/2011).

| **Vendor name** | |
| --- | --- |
| Aldrich CPR | Otava |
| Asinex | PBMR Labs |
| AsisChem | Pharmeks |
| ChemBridge | Princeton BioMolecular Research |
| ChemDiv | Ryan Scientific BB |
| Chemical Block | Specs |
| Enamine | TimTec |
| IBScreen | UORSY |
| Labotest | Vitas-M |
| Life Chemicals | NCI Plated 2007 |

**Table S2:** 2D structures of known inhibitors [[40](#_ENREF_66)].

| Structure | Structure | Structure | Structure | Structure |
| --- | --- | --- | --- | --- |
| 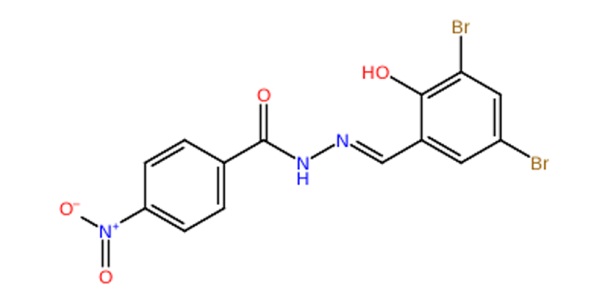  Compound1  Chemical formula: C_14_H_9_Br_2_N_3_O_4_ | 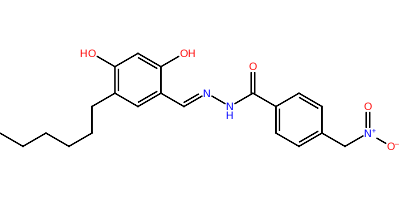  Compound2  Chemical formula: C_21_H_25_N_3_O_5_ | 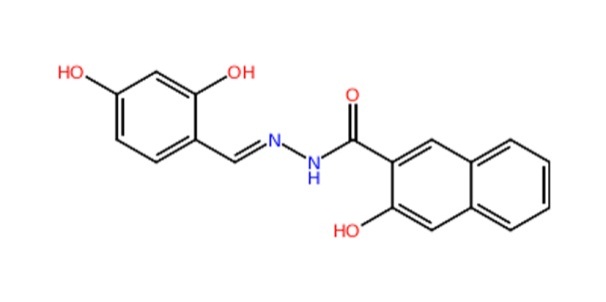  Compound3  Chemical formula: C_18_H_13_N_2_O_4_ | 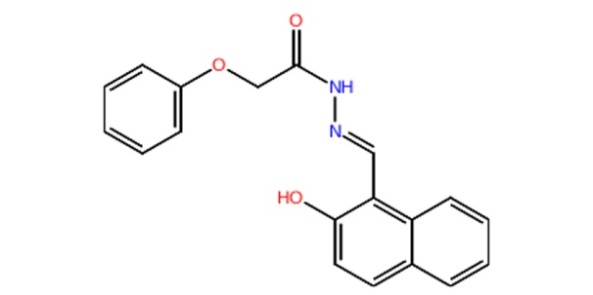  Compound4  Chemical formula: C_19_H_15_N_2_O_3_ | 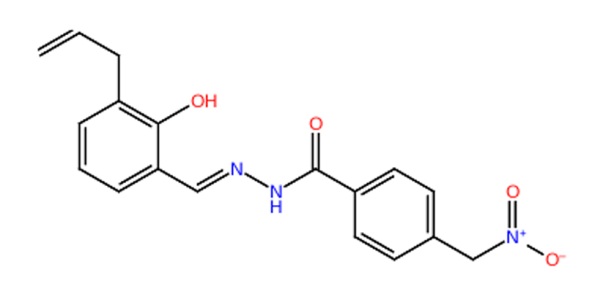  Compound5  Chemical formula: C_18_H_16_N_3_O_4_ |
| 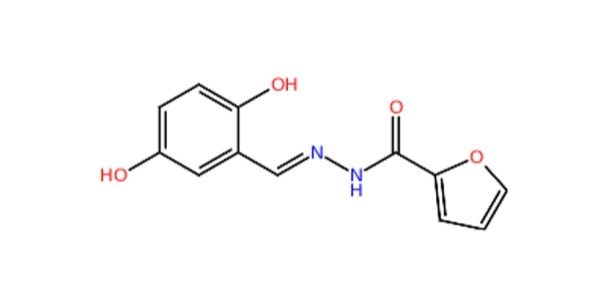  Compound6  Chemical formula: C_12_H_10_N_2_O_4_ | 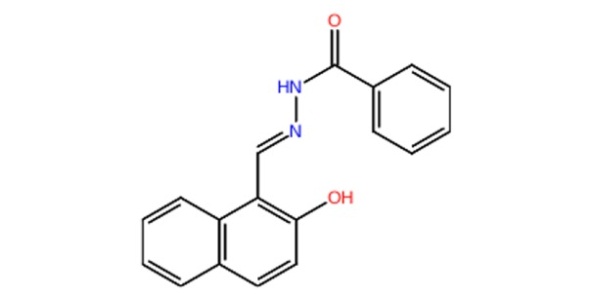  Compound7  Chemical formula: C_18_H_14_N_2_O_2_ | 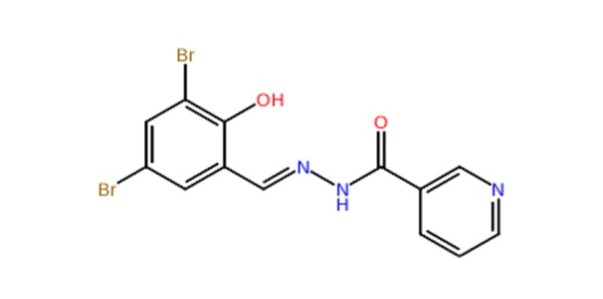  Compound8  Chemical formula: C_13_H_9_Br_2_N_3_O_2_ | 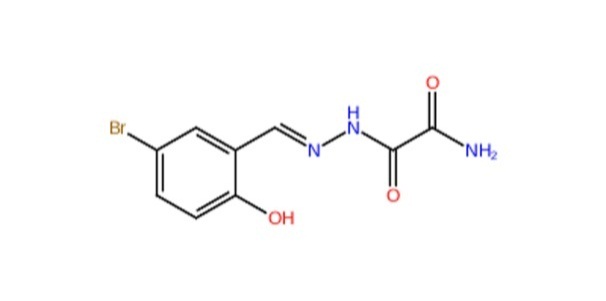  Compound9  Chemical formula: C_9_H_8_Br_1_N_3_O_3_ | 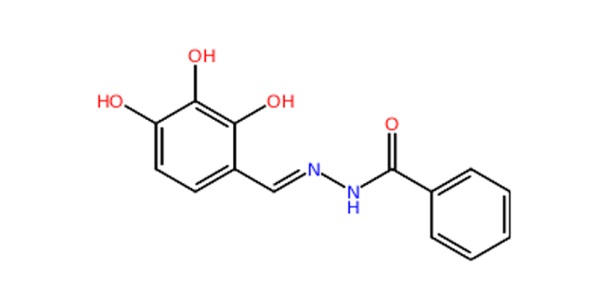  Compound10  Chemical formula: C_14_H_12_N_2_O_4_ |
| 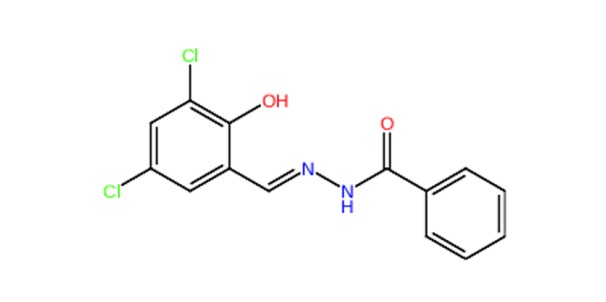  Compound11  Chemical formula: C_14_H_10_Cl_2_N_2_O_2_ | 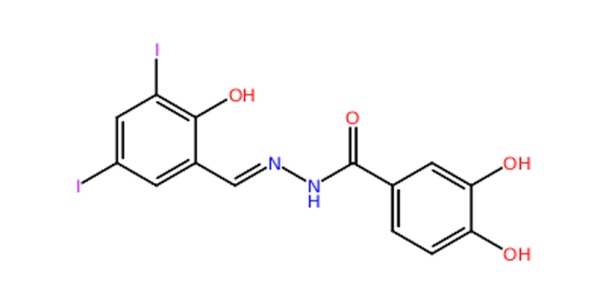  Compound12  Chemical formula: C_14_H_8_I_2_N_2_O_4_ | 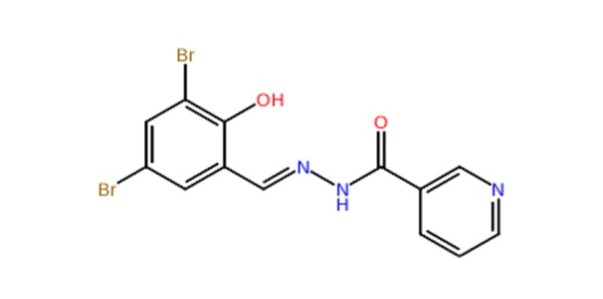  Compound13  Chemical formula: C_15_H_9_Br_2_N_3_O_4_ | 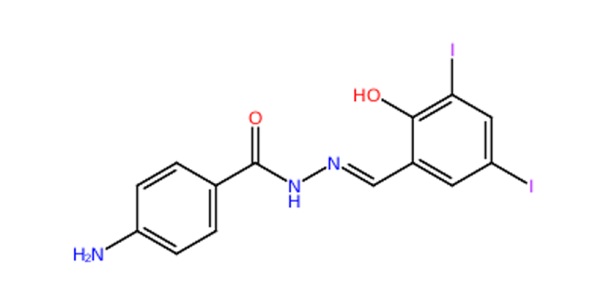  Compound14  Chemical formula: C_14_H_11_I_2_N_3_O_2_ | 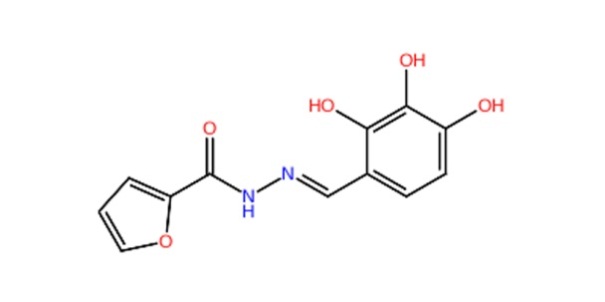  Compound15  Chemical formula: C_12_H_10_N_2_O_5_ |
| 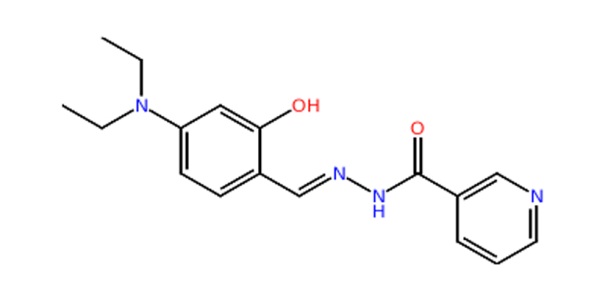  Compound16  Chemical formula: C_17_H_20_N_4_O_2_ | 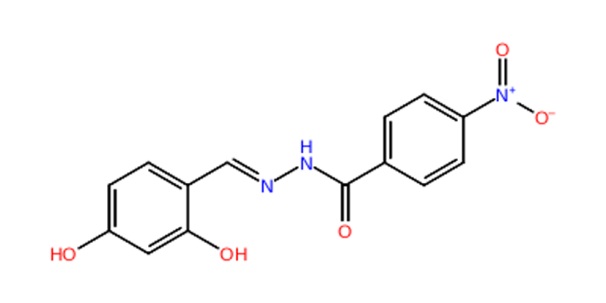  Compound17  Chemical formula: C_14_H_11_N_3_O_5_ | 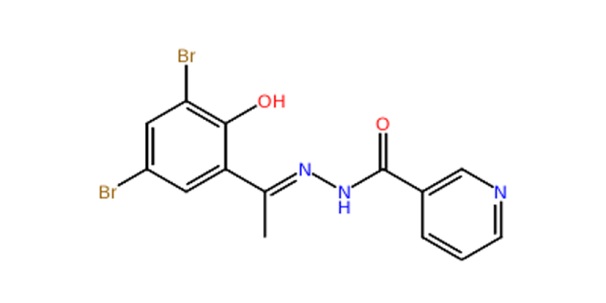  Compound18  Chemical formula: C_14_H_11_Br_2_N_3_O_2_ | 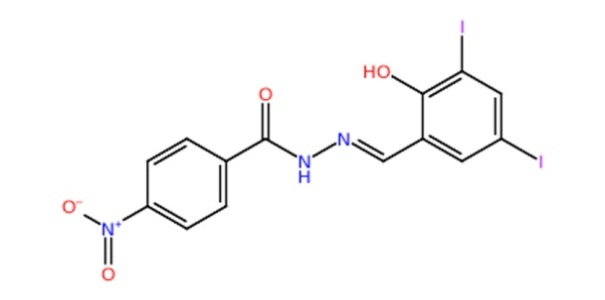  Compound19  Chemical formula: C_14_H_9_I_2_N_3_O_4_ | 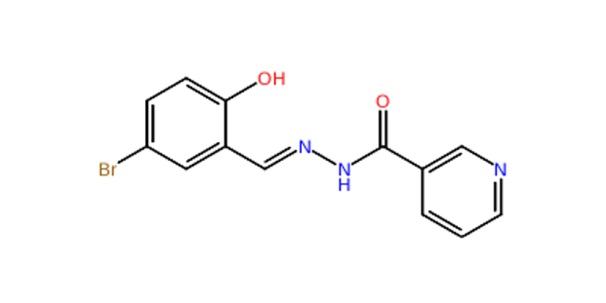  Compound20  Chemical formula: C_13_H_10_Br_1_N_3_O_2_ |
| ,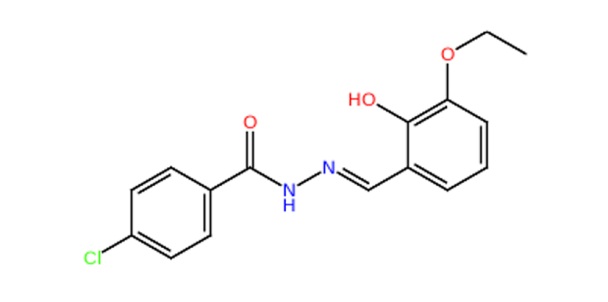  Compound21  Chemical formula: C_16_H_15_Cl_1_N_2_O_3_ | 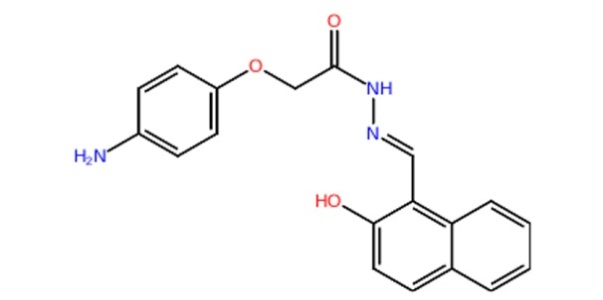  Compound22  Chemical formula: C_19_H_16_N_3_O_3_ | 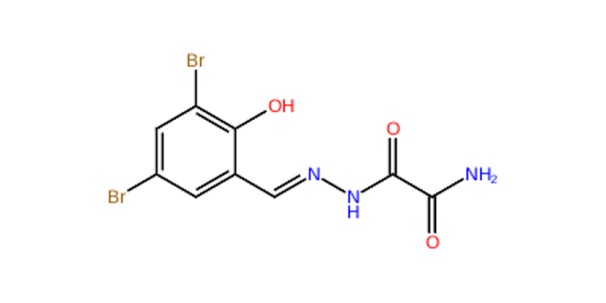  Compound23  Chemical formula: C_9_H_7_Br_2_N_3_O_3_ |  |  |

**Table S3:** AADDR.21 site point angle measurements.

| **Site1** | **Site2** | **Site3** | **Angle** |
| --- | --- | --- | --- |
| A2 | A1 | A4 | 171 |
| A2 | A1 | D10 | 81.2 |
| A2 | A1 | R12 | 148.4 |
| A4 | A1 | D10 | 89.8 |
| A4 | A1 | R12 | 40.6 |
| D10 | A1 | R12 | 130.4 |
| A1 | A2 | A4 | 5.4 |
| A1 | A2 | D10 | 38.7 |
| A1 | A2 | R12 | 18.2 |
| A4 | A2 | D10 | 33.3 |
| A4 | A2 | R12 | 23.6 |
| D10 | A2 | R12 | 56.9 |
| A1 | A4 | A2 | 3.6 |
| A1 | A4 | D10 | 25.6 |
| A1 | A4 | R12 | 61.2 |
| A2 | A4 | D10 | 22 |
| A2 | A4 | R12 | 64.8 |
| D10 | A4 | R12 | 86.8 |
| A1 | D10 | A2 | 60.1 |
| A1 | D10 | A4 | 64.6 |
| A1 | D10 | R12 | 32.8 |
| A2 | D10 | A4 | 124.7 |
| A2 | D10 | R12 | 92.9 |
| A4 | D10 | R12 | 31.8 |
| A1 | R12 | A2 | 13.4 |
| A1 | R12 | A4 | 78.2 |
| A1 | R12 | D10 | 16.8 |
| A2 | R12 | A4 | 91.6 |
| A2 | R12 | D10 | 30.2 |
| A4 | R12 | D10 | 61.4 |

**Table S4**: Database titles, docking scores and rankings of each member of all clusters, shown in kcal/mol.

| **Title** | **Docking score** | **Initial rank** | **Title** | **Docking score** | **Initial rank** |
| --- | --- | --- | --- | --- | --- |
| **cluster1** | | | **cluster2** | | |
| ZINC16525119 | -7.24 | 1 | ZINC17020779 | -6.35 | 6 |
| ZINC04982797 | -7.06 | 2 | ZINC17021043 | -6.22 | 7 |
| ZINC01663005 | -6.84 | 3 | ZINC01703513 | -6.21 | 8 |
| ZINC16545543 | -6.58 | 5 | ZINC17013669 | -6.05 | 9 |
| ZINC01581176 | -6.04 | 10 | ZINC17020456 | -5.93 | 13 |
| ZINC16545157 | -5.85 | 15 | ZINC17004909 | -5.85 | 14 |
| ZINC16545160 | -5.38 | 28 | ZINC17021040 | -5.81 | 16 |
| ZINC05599803 | -4.42 | 99 | ZINC01320041 | -5.72 | 19 |
| **cluster3** | | | ZINC16925199 | -5.72 | 20 |
| ZINC01205271 | -5.76 | 18 | ZINC16925192 | -5.70 | 21 |
| ZINC19800113 | -5.55 | 24 | ZINC16923221 | -5.68 | 22 |
| ZINC05297691 | -5.45 | 26 | ZINC17020453 | -5.41 | 27 |
| ZINC17747787 | -4.67 | 75 | ZINC03869837 | -5.26 | 32 |
| ZINC05125776 | -4.50 | 88 | ZINC01592668 | -4.96 | 51 |
| ZINC04637604 | -4.45 | 97 | ZINC04946348 | -4.90 | 55 |
| ZINC17970052 | -4.42 | 100 | ZINC17013428 | -4.84 | 57 |
| ZINC05297700 | -4.40 | 104 | ZINC00088349 | -4.80 | 59 |
| ZINC04637601 | -4.35 | 118 | ZINC17013666 | -4.78 | 61 |
| ZINC02387587 | -4.17 | 147 | ZINC04946582 | -4.75 | 65 |
| ZINC00230980 | -4.03 | 179 | ZINC17021036 | -4.73 | 66 |
|  |  |  | ZINC01669236 | -4.59 | 81 |
|  |  |  | ZINC04963105 | -4.47 | 92 |
|  |  |  | ZINC17020166 | -4.28 | 128 |
|  |  |  | ZINC09214236 | -4.11 | 154 |
|  |  |  | ZINC16931788 | -4.08 | 164 |
|  |  |  | ZINC17005625 | -4.04 | 175 |
|  |  |  | ZINC16925199 | -5.72 | 20 |

**Table S5:** Glide XP results of selected hits (receptor: YopE, hypothesis: AAADR.21). All values are shown in kcal/mol.

| **Title** | **Glide score** | **Glide lipo** | **Glide hbond** | **Glide**  **rewards** | **Glide evdw** | **Glide ecoul** | **Glide rotb** | **Glide**  **esite** | **Glide**  **emodel** |
| --- | --- | --- | --- | --- | --- | --- | --- | --- | --- |
| ZINC16525119 | -7.24 | -0.5044 | -0.6757 | -1.9077 | -4.42 | -23.79 | 0.9121 | 0.0000 | -30.99 |
| ZINC04982797 | -7.06 | -0.7608 | -0.1398 | -1.0625 | -11.55 | -19.15 | 0.8014 | -0.0709 | -39.41 |
| ZINC01663005 | -6.84 | -0.5550 | -0.1102 | -1.6961 | -2.33 | -21.46 | 0.8329 | -0.0778 | -23.80 |
| ZINC17020779 | -6.35 | -1.4097 | -0.2927 | -1.4138 | -11.57 | -16.57 | 0.7653 | -0.0147 | -32.71 |
| ZINC17021043 | -6.22 | -0.9839 | -0.4021 | -1.3886 | -6.13 | -15.94 | 0.4172 | -0.0512 | -27.33 |
| ZINC01703513 | -6.21 | -1.1692 | -0.7362 | -1.5028 | -7.41 | -18.16 | 0.9026 | -0.0276 | -32.82 |
| ZINC01205271 | -5.76 | -1.2287 | -0.8793 | -1.0292 | -14.43 | -15.73 | 0.8773 | -0.0004 | -38.36 |
| ZINC19800113 | -5.55 | -0.9856 | -0.5321 | -1.0993 | -18.69 | -17.45 | 0.8773 | 0.0000 | -40.66 |
| ZINC05297691 | -5.45 | -0.7924 | -1.1734 | -1.3985 | -11.16 | -16.52 | 1.0230 | -0.0800 | -32.16 |

Lipo: lipophilic energy

Hbond: hydrogen bonding energy

Evdw: van der Waals energy

Ecoul: coulomb energy

RotB: rotatable bond penalty

Esite: non-hydrogen-bonding polar interaction energy

**Table S6:** ADME and pharmacokinetic properties of the selected hits. All properties are determined by QikProp.

| **Title** | **#stars** | **#amine** | **#amidine** | **#acid** | **#amide** | **#rotor** | **#rtvFG** | **CNS** | **mol MW** | **dipole** | **SASA** | **FOSA** | **FISA** | **PISA** | **WPSA** | **volume** | **donorHB** |
| --- | --- | --- | --- | --- | --- | --- | --- | --- | --- | --- | --- | --- | --- | --- | --- | --- | --- |
| ZINC16525119 | 0 | 0 | 0 | 0 | 0 | 8 | 0 | -2 | 238.2 | 6.13 | 470.824 | 81.664 | 196.849 | 192.311 | 0 | 774.27 | 5 |
| ZINC04982797 | 0 | 0 | 0 | 0 | 0 | 8 | 0 | -2 | 344.2 | 4.775 | 546.696 | 83.187 | 191.565 | 194.535 | 77.409 | 916.442 | 4 |
| ZINC01663005 | 0 | 0 | 0 | 0 | 0 | 8 | 0 | -2 | 251.2 | 6.43 | 481.916 | 80.009 | 214.371 | 187.537 | 0 | 798.993 | 4 |
| ZINC17020779 | 0 | 0 | 0 | 0 | 0 | 7 | 1 | -2 | 311.3 | 4.104 | 573.569 | 338.628 | 177.27 | 57.671 | 0 | 995.819 | 3 |
| ZINC17021043 | 0 | 0 | 0 | 0 | 0 | 5 | 0 | -1 | 296.3 | 7.035 | 499.093 | 181.302 | 139.702 | 153.145 | 24.944 | 874.045 | 3 |
| ZINC01703513 | 0 | 0 | 0 | 0 | 0 | 7 | 1 | -2 | 283.3 | 5.624 | 502.53 | 164.196 | 182.498 | 155.836 | 0 | 870.749 | 3 |
| ZINC01205271 | 0 | 0 | 0 | 0 | 0 | 10 | 0 | -2 | 362.4 | 7.661 | 704.149 | 428.825 | 179.792 | 95.533 | 0 | 1195.846 | 3 |
| ZINC19800113 | 0 | 0 | 0 | 0 | 0 | 10 | 0 | -2 | 362.4 | 8.026 | 680.707 | 394.813 | 180.424 | 105.47 | 0 | 1176.894 | 3 |
| ZINC05297691 | 0 | 0 | 0 | 0 | 0 | 9 | 0 | -2 | 332.4 | 9.632 | 655.767 | 327.526 | 179.761 | 148.48 | 0 | 1111.162 | 3 |
| **Title** | **accptHB** | **dip^2/V** | **ACxDN^.5/SA** | **glob** | **QPpolrz** | **QPlogPC16** | **QPlogPoct** | **QPlogPw** | **QPlogPo/w** | **QPlogS** | **CIQPlogS** | **QPlogHERG** | **QPPCaco** | **QPlogBB** | **QPPMDCK** | **QPlogKp** | **IP(eV)** |
| ZINC16525119 | 8.3 | 0.048535 | 0.039419 | 0.866099 | 21.532 | 9.214 | 19.297 | 17.233 | -0.866 | -1.488 | -1.703 | -4.616 | 134.641 | -1.668 | 56.636 | -3.702 | 8.54 |
| ZINC04982797 | 8.8 | 0.024876 | 0.032193 | 0.83462 | 27.243 | 10.807 | 20.08 | 16.45 | 0.414 | -2.616 | -3.39 | -5.136 | 151.108 | -1.562 | 170.335 | -3.597 | 9.089 |
| ZINC01663005 | 9.8 | 0.051743 | 0.040671 | 0.864084 | 22.476 | 9.318 | 19.105 | 17.359 | -1.023 | -1.398 | -1.553 | -4.626 | 91.838 | -1.833 | 37.454 | -4.042 | 9.497 |
| ZINC17020779 | 8.8 | 0.016911 | 0.026574 | 0.840814 | 29.755 | 9.775 | 18.79 | 14.215 | 0.626 | -2.791 | -2.586 | -4.383 | 206.468 | -1.53 | 89.905 | -3.911 | 8.387 |
| ZINC17021043 | 8.3 | 0.056616 | 0.028804 | 0.885808 | 27.077 | 9.37 | 18.393 | 14.387 | 0.641 | -2.298 | -2.669 | -4.11 | 468.928 | -0.864 | 298.881 | -3.075 | 8.427 |
| ZINC01703513 | 8.8 | 0.036327 | 0.030331 | 0.877536 | 25.687 | 9.436 | 17.9 | 14.747 | 0.055 | -1.82 | -2.11 | -4.257 | 184.194 | -1.438 | 79.469 | -3.662 | 8.626 |
| ZINC01205271 | 5.8 | 0.049076 | 0.014144 | 0.773779 | 36.195 | 12.031 | 19.596 | 11.138 | 2.892 | -5.131 | -4.817 | -5.648 | 195.405 | -2.041 | 84.71 | -3.536 | 8.44 |
| ZINC19800113 | 5.8 | 0.054733 | 0.014631 | 0.791948 | 35.532 | 11.875 | 19.536 | 11.154 | 2.797 | -4.736 | -4.817 | -5.401 | 192.727 | -1.957 | 83.456 | -3.513 | 8.731 |
| ZINC05297691 |  |  |  |  |  |  |  |  |  |  |  |  |  |  |  |  |  |
| **Title** | **EA(eV)** | **#metab** | **QPlogKhsa** | **HOA** | **%HOA** | **SAfluorine** | **SAamideO** | **PSA** | **#NandO** | **RuleOfFive** | **RuleOfThree** | **#ringatoms** | **#in34** | **#in56** | **#noncon** | **#nonHatm** | **Jm** |
| ZINC16525119 | 0.005 | 4 | -0.908 | 2 | 59.986 | 0 | 0 | 111.071 | 6 | 0 | 0 | 9 | 0 | 9 | 0 | 17 | 1.785549 |
| ZINC04982797 | 0.792 | 4 | -0.758 | 3 | 68.376 | 0 | 0 | 113.268 | 7 | 0 | 0 | 11 | 0 | 11 | 0 | 20 | 0.210863 |
| ZINC01663005 | 1.572 | 4 | -0.999 | 2 | 56.092 | 0 | 0 | 122.252 | 7 | 0 | 0 | 10 | 0 | 10 | 0 | 18 | 0.962855 |
| ZINC17020779 | 0.304 | 7 | -0.544 | 2 | 72.042 | 0 | 0 | 119.1 | 7 | 0 | 1 | 11 | 0 | 11 | 4 | 22 | 0.061843 |
| ZINC17021043 | 0.081 | 4 | -0.582 | 3 | 78.506 | 0 | 0 | 88.247 | 6 | 0 | 0 | 14 | 0 | 14 | 4 | 20 | 1.255382 |
| ZINC01703513 | 0.323 | 6 | -0.744 | 3 | 67.814 | 0 | 0 | 119.559 | 7 | 0 | 0 | 11 | 0 | 11 | 4 | 20 | 0.934124 |
| ZINC01205271 | 0.466 | 7 | 0.186 | 2 | 84.881 | 0 | 0 | 124.12 | 9 | 0 | 1 | 11 | 0 | 11 | 0 | 26 | 0.000779 |
| ZINC19800113 | 0.576 | 7 | 0.152 | 2 | 84.219 | 0 | 0 | 124.045 | 9 | 0 | 1 | 11 | 0 | 11 | 0 | 26 | 0.002044 |
| ZINC05297691 | 0.561 | 6 | 0.142 | 3 | 83.911 | 0 | 0 | 116.681 | 8 | 0 | 0 | 11 | 0 | 11 | 0 | 24 | 0.002513 |

**
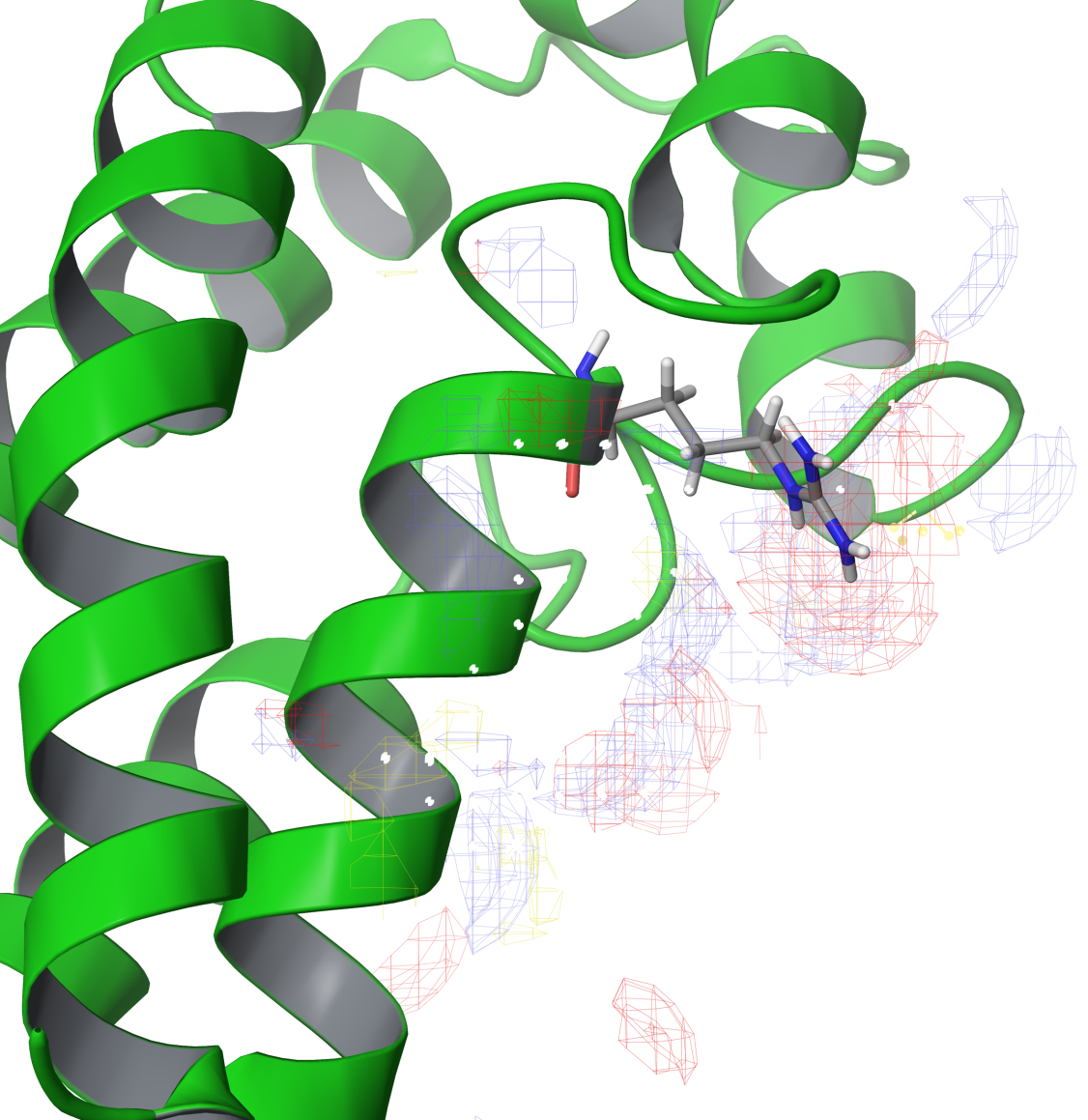
**

**Figure S1:** SiteMap output of YopE. Green grids denote hydrophilic sites whereas yellow grids denote hydrophobic sites, blue grids denote hydrogen bond donors and red grids denote hydrogen bond acceptors. The critical Arg144 residue is shown as stick.

**
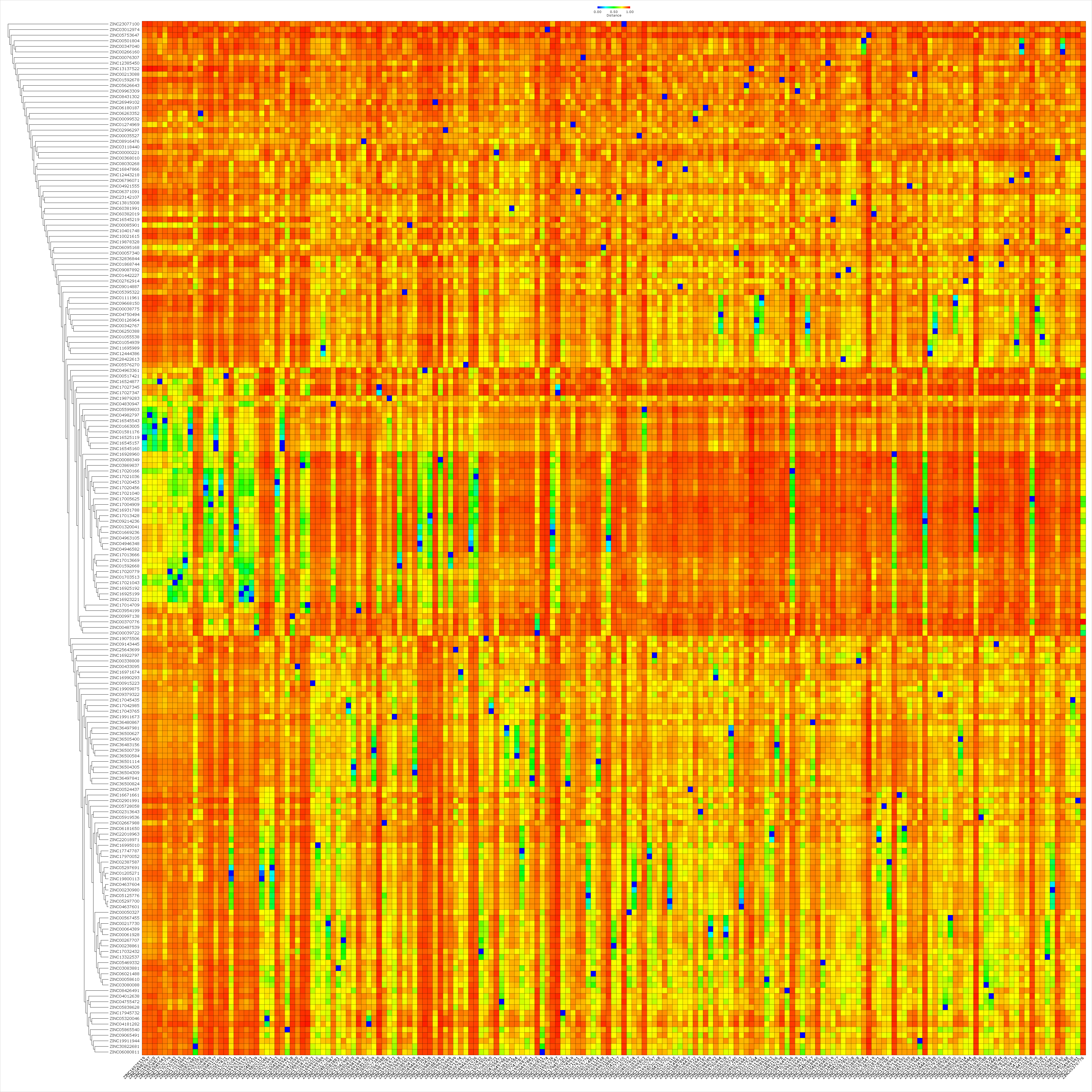
**

**Figure S2:** ChemMine hierarchical similarity tree of top 185 hits with distance matrix calculate by single linkage method.


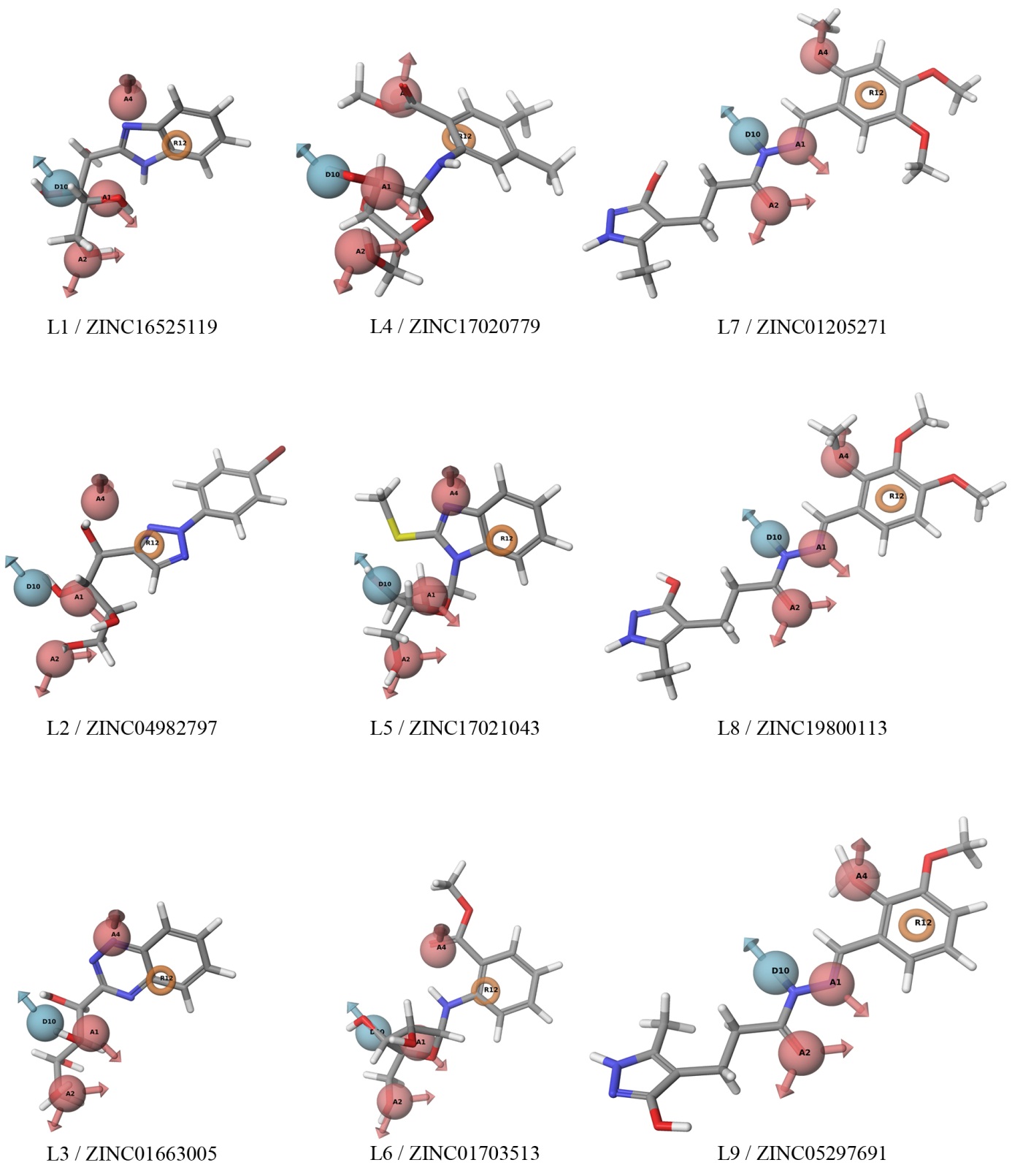


**Figure S3:** AAADR.21 pharmacophore site point superposition to the selected hits.
